# Unravelling the Carbon and Sulphur Metabolism in Coastal Soil Ecosystems Using Comparative Cultivation-Independent Genome-Level Characterisation of Microbial Communities

**DOI:** 10.1371/journal.pone.0107025

**Published:** 2014-09-16

**Authors:** Basit Yousuf, Raghawendra Kumar, Avinash Mishra, Bhavanath Jha

**Affiliations:** 1 Discipline of Marine Biotechnology and Ecology, CSIR-Central Salt and Marine Chemicals Research Institute, Bhavnagar, Gujarat, India; 2 Academy of Scientific and Innovative Research, CSIR, New Delhi, India; Wilfrid Laurier University, Canada

## Abstract

Bacterial autotrophy contributes significantly to the overall carbon balance, which stabilises atmospheric CO_2_ concentration and decelerates global warming. Little attention has been paid to different modes of carbon/sulphur metabolism mediated by autotrophic bacterial communities in terrestrial soil ecosystems. We studied these pathways by analysing the distribution and abundance of the diagnostic metabolic marker genes *cbbM*, *apsA* and *soxB*, which encode for ribulose-1,5-bisphosphate carboxylase/oxygenase, adenosine phosphosulphate reductase and sulphate thiohydrolase, respectively, among different contrasting soil types. Additionally, the abundance of community members was assessed by quantifying the gene copy numbers for 16S rRNA, *cbbL*, *cbbM*, *apsA* and *soxB*. Distinct compositional differences were observed among the clone libraries, which revealed a dominance of phylotypes associated with carbon and sulphur cycling, such as *Gammaproteobacteria* (*Thiohalomonas*, *Allochromatium, Chromatium, Thiomicrospira*) and *Alphaproteobacteria* (*Rhodopseudomonas, Rhodovulum, Paracoccus*). The rhizosphere soil was devoid of sulphur metabolism, as the *soxB* and *apsA* genes were not observed in the rhizosphere metagenome, which suggests the absence or inadequate representation of sulphur-oxidising bacteria. We hypothesise that the novel *Gammaproteobacteria* sulphur oxidisers might be actively involved in sulphur oxidation and inorganic carbon fixation, particularly in barren saline soil ecosystems, suggesting their significant putative ecological role and contribution to the soil carbon pool.

## Introduction

Soil microbial communities are indispensable for the health of the Earth as they drive major biogeochemical cycles, play a critical role in agriculture and have a significant impact on climatic change [1]–[2]. Autotrophic soil microorganisms are an integral component of the ecosystem and facilitate the availability of otherwise unavailable CO_2_ to other organisms. This assimilation process occurs through various complex biochemical pathways [3]. The distribution of carbon fixation strategies are widespread among prokaryotes and depends on the individual autotrophic organism and is also determined by different habitat characteristics, such as the energy demand, the availability of inorganic compounds (sulphide, elemental sulphur, thiosulphate and sometimes ferrous iron and hydrogen), usage of coenzymes and the oxygen sensitivity of enzymes [4].

The predominant route of this fixation process is the Calvin–Benson–Bassham (CBB) reductive pentose phosphate pathway, which is ubiquitously prevalent among the aerobic members of the *Alpha*-, *Beta*- and *Gammaproteobacteria* [5]. The key enzyme of this pathway, ribulose 1,5-bisphosphate carboxylase/oxygenase (RuBisCO), occurs in forms I and II, whose large subunits are encoded by the *cbbL* and *cbbM* genes, respectively. The *cbbL* gene is found in plants, green algae, *Cyanobacteria* and many chemolithoautotrophs, whereas *cbbM* is reported to occur in several photosynthetic bacteria, aerobic and facultative anaerobic chemoautotrophic bacteria and dinoflagellates [6]. The occurrence of the *cbbM* gene has been exclusively investigated for chemolithoautotrophy from aquatic habitats such as hydrothermal vents [5], hypersaline habitats [7], soda lake sediments [8], thermal Springs [9], Movile Cave in Romania [10], with only one study from a terrestrial ecosystem reported so far [11].

Microbial sulphur oxidation is one of the vital processes for the biogeochemical sulphur cycle and is closely linked to carbon cycling. The sulphur-based assimilation of inorganic carbon occurs via complex sulphur oxidation mechanisms. One pathway involves the complete oxidation of reduced sulphur compounds to sulphate, which occurs in the SOX pathway, whereas the APS pathway implicates adenosine-5-phosphosulphate as an intermediate [12]. The key genes include those encoding adenosine-5-phosphosulphate, reductase alpha-subunit (*apsA*) and thiosulphate-oxidizing complex (*sox*). The Sox pathway operates in a wide range of photo- and chemoautotrophic bacteria [13]. A fully functional Sox complex involves SoxB, SoxXA, SoxYZ and SoxCD components, among which SoxB is considered to be the key constituent [12]. The *soxB* gene is regarded as a useful phylogenetic marker and its presence has been demonstrated in various environments, particularly from aquatic habitats such as marine sediments, hydrothermal vents and soda lakes [13]–[15]. The *apsA* gene can reveal the occurrence of both sulphate-reducing prokaryotes as well as sulphur-oxidising prokaryotes [16]–[17], which have been investigated in different environments, such as hydrothermal vents, saline alkaline soil and Qinghai-Tibetan Lakes [15], [18]–[19]. Sulphur-oxidising bacterial communities encompass physiologically and phylogenetically diverse members of *Alpha*-, *Beta*-, *Gamma-* and *Epsilonproteobacteria*, *Chlorobia* and *Chloroflexi* [12]. The sulphur-reducing bacteria mostly belong to *Deltaproteobacteria* [20]–[21].

This study represents the first comparative molecular analysis of metabolic marker genes (*cbbM*, *apsA* and *soxB*) performed by targeted metagenomics of key enzymes of different complex biochemical pathways involved in autotrophy in coastal saline, agricultural and rhizosphere soil niches. This study is complementary to a previously performed determination of community structure at these sites based on 16S rRNA and *cbbL* genes [22]–[23]. The aim of the present work was to broaden our view on the diversity and abundance of alternative modes of autotrophic metabolism.

## Experimental Procedures

### Ethics Statement

Sampling locations are not the part of any national parks or protected areas and do not require any specific permits. It is further to confirm that the field studies did not involve endangered or protected species and the specific location of sampling sites was given with their respective description.

### Soil samples and physicochemical characteristics

The study was conducted on three bulk soil types (high saline, low saline and agriculture) and one rhizosphere soil type, situated along the Arabian Sea coast, Gujarat, India. There was no crop in the agricultural field at the time of sample collection. However, farmers grew cotton and groundnut regularly in the field. The bulk soil types (0–10 cm of topsoil) were collected from nine transects of each site and sieved through 2 mm mesh to make three composite soil samples per site. The rhizosphere sample was taken from nine randomly selected plantlets, by separating soil tightly adhered to the roots to make three composite rhizosphere samples. The four sampling sites were designated as (i) SS1- saline soil samples collected from the barren land away from the sea coast (N 21°35.711′, E 72°16.875′); (ii) SS2- saline soil samples collected from barren land near the sea coast (N 21°45.402′, E 72°14.156′); (iii) AS- soil samples collected from the agricultural field (N 20°53.884′, E 70°29.730′); (iv) RS- soil samples collected from the rhizosphere (N 20°53.884′, E 70°29.730′). These soil samples were transported to the laboratory immediately and frozen at −20°C for further processing. The composite soil samples were imperilled to physical and chemical analyses for determining the major soil characteristics. The soil pH and salinity were measured on air-dried soil in deionised water by using a 1∶2 (w/v) soil:liquid ratio using the Seven Easy pH and Conductivity meter (Mettler-Toledo AG, Switzerland). The total soil organic carbon was analysed by Liqui TOC (Elementar, Germany) while total carbon, nitrogen and sulphur contents were determined by CHNS analyser (Perkin Elmer series ii, 2400, USA).

### Metagenome extraction and PCR amplification

Total soil DNA was extracted in triplicate from 500 mg of each soil samples [22]–[23] and was used as template to amplify targeted genes (*cbbM*, *apsA*, *aclB* and *soxB*) using gene specific primers following their respective PCR conditions (**Table S1**).

### Generation of clone libraries

The functional genes (*cbbM*, *apsA* and *soxB*) were amplified individually from each site (in triplicate). The products were processed by excising expected amplicon size from the gel, purified by using QIAquick gel extraction kit (Qiagen, Hilden, Germany) and cloned into the pGEM-T/pGEM-T Easy vector (Promega, Madison, WI, USA). Clones were selected randomly, screened for the presence of correct inserts sizes (520, 380 and 753 of *cbbM*, *apsA* and *soxB* genes, respectively) and the positive clones were sequenced (at M/s Macrogen Inc., S. Korea).

### Alignment and phylogenetic reconstruction

The nucleotide sequences showing anomalous short or longer lengths and poor quality, and chimeric sequences were removed from the data to eliminate the inaccuracy in assessment of community structure. The taxonomic affiliation of these functional genes was assessed by using a BLASTn identity and BLASTx, similarity but the affiliations were given based on BLASTn identity search tool.

The multiple nucleotide sequence alignment was performed using Clustal Omega to estimate the number of representative operational taxonomic units (OTUs), generated using Mothur program [24]. The evolutionary history of all the genes was inferred by the Maximum Likelihood method of by *MEGA* v.5.2 using bootstrap resampling method with 500 bootstrap replications [25]. Model selection analysis was conducted to calculate the best-fit model of nucleotide substitution by *MEGA* v.5.2 based on lowest Bayesian Information Criterion [25]. The codon positions included were 1st+2nd+3rd+Noncoding and the positions containing gaps and missing data were eliminated from the datasets.

### Community structure determination based on functional genes

The threshold for OTUs generally varies amongst different genes. Total four thresholds 92, 95, 97 and 99% nucleotide sequence identity were tested (data not shown) to cluster nucleotide sequences into operational taxonomic units (OTUs). Among these, sequence identity cut-off of 95% was used further in the study to define an OTU. It uses the furthest neighbour method to assort similar sequences into groups at arbitrary levels of taxonomic identity. Multiple sequence alignment was performed with Clustal Omega. The Jukes-Cantor evolutionary distance matrices were calculated by the DNADIST program within the PHYLIP [26]. Rarefaction curves, coverage richness estimators (Chao and ACE) and diversity indices (Shannon and Simpson index) were determined using Mothur [24].

### Assessment of environmental clustering and statistical analysis

The rooted phylogenetic tree was generated and imported into UniFrac along with the environmental labels [27]. Phylogenetic tree based analysis of community diversity was performed using the UniFrac significance test and P test. The *P* tests were also corrected for multiple comparisons (Bonferonni correction) which indicate phylogenetic distribution between samples by pairwise comparisons and also determine whether environments are significantly different.

The relationships between the major taxonomic groups and environmental factors (e.g. pH, Electrical Conductivity- EC, Total Carbon- TC, Total Nitrogen- TN and Total Sulphur- TS) were analysed by stepwise canonical correspondence analysis (CCA) using PAST [28]. Hierarchical clustering of physicochemical data was built with complete linkage method using euclidean distances between data points. Permutation tests were carried out using two-way ANOVA for all analyses and *P* value of 0.05 (*P≤0.05*) was considered significant.

### Quantitative real time-PCR (qRT-PCR)

Gene copy number (of 16S rRNA, *cbbL*, *cbbM*, *apsA*, and *soxB* per g of soil) was enumerated by qRT PCR using gene specific primers and standardised annealing temperatures [22] (**Table S1**). The experiments were repeated three times independently.

### GenBank submission and accession numbers

All the validated nucleotide sequence data reported in this study were deposited in the GenBank database with accession numbers as KF788311-KF788755- *cbbM* gene sequences; KF788756-KF789143- *apsA* gene sequences; KF789144-KF789459- *soxB* gene sequences from all clone libraries.

## Results

Soils collected from four different sites: three bulk soil types (SS1-low saline, SS2-high saline, AS-agricultural) and a rhizosphere soil (RS), showed variations in water content, pH, salinity, organic carbon, nitrogen and sulphur contents (**Table S2**). Sites were selected to understand the occurrence of different biochemical pathways in these ecologically distinct niches and to compare bulk and rhizosphere soil types.

### Functional communities based on the *cbbM* gene

The *cbbM* clone libraries resulted in 102, 113, 101 and 129 clone sequences from SS1, SS2, AS and RS respectively. These *cbbM* clone sequences were grouped into 44, 35, 16 and 16 unique OTUs (phylotypes) within their respective clone libraries (**Table 1**). The low richness of *cbbM* genes was detected as 0.12 and 0.15 OTU per clone for RS and AS clone libraries, however, it was higher for SS1 and SS2 (0.43, 0.30 OTU per clone). The most dominant phylotypes of the SS1 clone library showed affiliation to *Gammaproteobacteria* (46 clones), *Rhodopseudomonas palustris* (18 clones) and *Thiohalorhabdus denitrificans* (nine clones); fourteen clones were related to uncultured bacteria. Other phylotypes were allied to numerous genera, which were represented by fewer clones. The majority of clones from the SS2 library were attributed to RuBisCO genes from *Thiohalomonas denitrificans* (22 clones), *Rhodopseudomonas palustris* (17 clones), *Thiohalomonas nitratireducens* (15 clones) and *Rhodovulum sulfidophilum* (five clones). The second-largest group comprised uncultured bacterium consisting of twenty six clones. *Magnettospirillium magnetotacticum, Gammaproteobacteria* and *Halothiobacillus* were the other genera represented in this library. The agricultural soil was dominated by clones associated with *Rhodopseudomonas palustris* (22 clones), *Rhodovulum sulfidophilum* (17 clones) and *Thiobacillus denitrificans* (nine clones). The maximum number of clones was assigned to *Gammaproteobacteria* (53 clones). The soil rhizosphere was exclusively dominated by *Gammaproteobacteria* (103 clones) followed by *Thiohalomonas nitratireducens* (17 clones). Although all the *cbbM* clone libraries consisted primarily of *Gammaproteobacteria* and *Alphaproteobacteria* phylogenetic groups, the relative abundance of the functional microbial groups varied considerably (**Figure S1**). Most nucleotide sequences displayed 75 to 88% sequence identity to their closest relatives according to a BLASTn identity search, but some *cbbM* genes showed greater sequence identity (91–96%) and were related to *Thiohalomonas denitrificans, Thiobacillus denitrificans* and uncultured clones. We also tested the occurrence of the *aclB* gene using different PCR conditions, but were unable to amplify it, suggesting it is rare or absent (**Figure S2**).

**Table 1:** Biodiversity and predicted richness of the *cbbM, apsA* and *soxB* gene sequences.

Genes	No of clones	Sobs^1^ (OTU)	Shannon Weiner (H)	Simpson (1-D)	Chao	ACE	Jackknife	Coverage (%)	No of Singletons
*cbbM*									
SS1	102	44	3.0	0.90	89.1	167.0	94.8	71	28
SS2	113	35	2.6	0.86	50.5	93.5	54	84	18
AS	101	16	2.02	0.82	21.2	42.1	23	93	29
RS	129	16	1.5	0.62	23	50.3	24	93	8
*apsA*									
SS1	117	46	3.1	0.90	113.7	129.5	159.8	75	29
SS2	140	69	3.8	0.97	151.5	269.8	160.4	67	79
AS	131	53	3.4	0.96	97	215.2	92.4	73	34
*soxB*									
SS1	107	35	3.0	0.91	43.3	49.5	49	86	14
SS2	117	47	3.4	0.97	101	147.6	102.6	75	28
AS	93	46	3.6	0.98	59.1	67.7	67	77	21

### Functional community based on the *apsA* gene

To investigate the distribution of sulphur-oxidising photo- and chemoautotrophs, we established the *apsA* clone libraries from SS1, SS2 and AS, which comprised 117, 140, 131 valid sequences. These sequences could be grouped into 46, 69 and 53 phylotypes, respectively (**Table 1**). Despite of repeated attempts to amplify the *apsA* gene from the metagenome of rhizospheric soil using previously described and various modified PCR conditions, the gene could not be amplified, which revealed the absence or inadequate representation of sulphur-oxidising bacteria (**Figure S2**). These three habitats were characterised by phylotypes allied to few cultured signature genera, which totally dominated the community, for example: *Chromatium okenii* (SS1, SS2, AS; 3, 3, 39 clones), *Allochromatium minutissimum* (5, 2, 33 clones) exclusively dominated at the agricultural site; similarly, *Deltaproteobacteria*- *Desulfarculus baarsii, Desulfococcus oleovorans, Desulfovibrio giganteus, Desulfofustis glycolicus, Desulfomicrobium baculatum* (6, 3, 0 clones), *Gammaproteobacteria* (1, 17, 1 clones), *Robbea* (3, 18, 1 clones), *Olavius algarvensis* (9, 5, 0 clones), *Thiobacillus plumbophilus, T. thioparus, T. denitrificans* (6, 5, 6 clones), *Thiochromatium tepidum* (1, 2, 2 clones), *Halochromatium glycolicum* (0, 1, 1 clones) and *Thiococcus pfennigii* (0, 7, 2 clones) dominated in saline soil ecosystems. Uncultured bacteria (76, 53, 43 clones) were the most dominant group and were represented all habitats. The other saline soil clone libraries were affiliated to genera, represented by fewer clones. The levels of nucleotide sequence identity ranged from 71 to 90% for most clone sequences. A few clones showed a nucleotide identity up to 93% and one clone from the SS1 library displayed 100% nucleotide identity with an uncultured *Gammaproteobacterium* clone isolated from the gut microflora, reported from Vanuatu.

### Functional community based on the *soxB* gene

To further analyse the potential of sulphur oxidation at these four sites, the *soxB* gene was amplified from the SS1, SS2 and AS metagenomes. The lack of amplification in the RS metagenome further confirmed the absence or inadequate representation of sulphur-oxidising bacteria (**Figure S2**). The *soxB* gene clone libraries resulted in 107, 117 and 93 valid sequences which were grouped into 35, 47 and 46 phylotypes, respectively (**Table 1**). The SS1 clone library was represented by phylotypes allied to *Rhodothalassium salexigens* (15 clones), *Thiomicrospira crunogena* (18 clones), *Paracoccus* (12) and a further 12 clones were related to uncultured bacteria. A large number of clones (30) were related to Endosymbionts of *Ifremeria* (74% sequence identity). The SS2 clone library consisted of dominant phylotypes that were ascribed to genera such as *Spirochaeta* (25 clones), *Rhodovulum adriaticum* (17 clones) and *Thiomicrospira crunogena* (11 clones). A total of thirty three clones showed no affiliation to any recognised cultured genera. The other bacterial groups were similar to those in SS1, except for *Hydrogenophaga* (2 clones), *Azospirillum* (1 clone), sulphur-oxidising bacteria (1 clone), *Thiohalomonas denitrificans* (1 clone) and *Pandoraea* (3 clones). The agricultural soil clone library was not dominated by any particular group, but had an equal distribution of numerous bacterial genera which differed from those in saline soil clone libraries and include: *Marichromatium purpuratum* (8 clones), *Thiocystis violacea* (5 clones), *Thiocapsa roseopersicina*, *Thiobacillus denitrificans* and *Thiobacillus aquaesulis*. These bacterial genera belonged to *Gammaproteobacteria*, *Betaproteobacteria* and a few clones were related to *Alphaproteobacteria* phylogenetic groups (**Figure S1**). Most phylotypes were related to uncultured clones (28) and showed 70 to 85% sequence identity to their closest relatives according to BLASTn identity searches. Two clones from the AS library exhibited 100% nucleotide sequence identity with uncultured clones reported from coastal sediments from Janssand, Germany.

### Comparative molecular phylogeny

**Figure 1:**
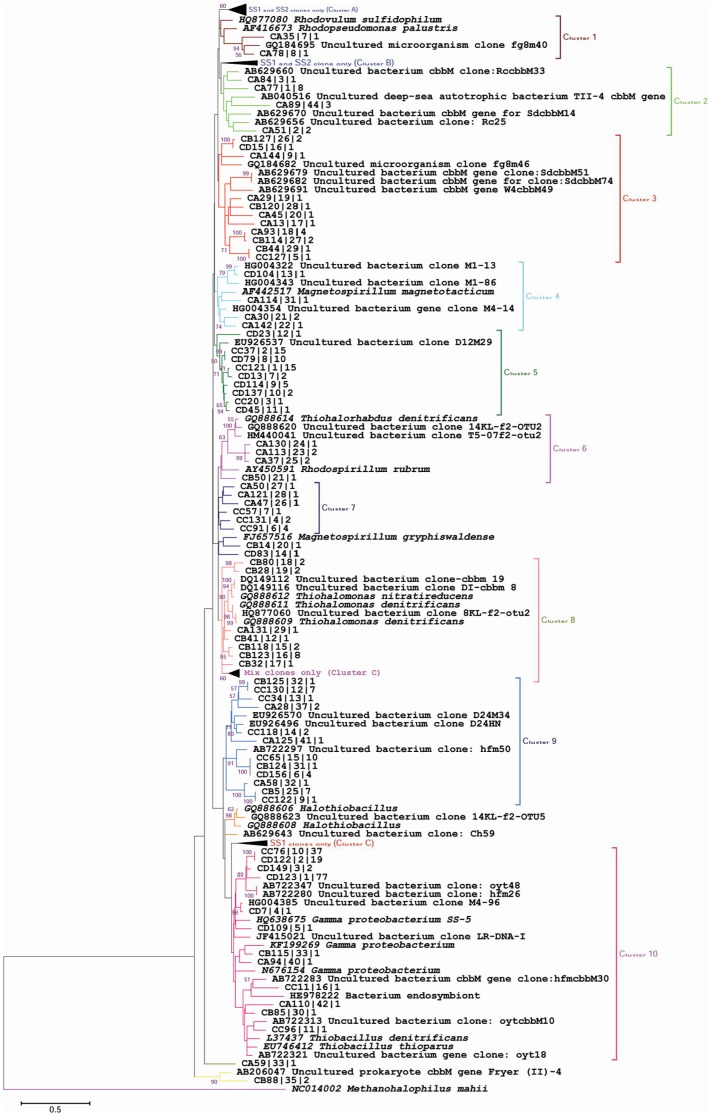
Phylogenetic analysis of the *cbbM* gene clones. The final dataset of 168 nucleotide sequences include sequences from low saline (SS1), high saline (SS2), agricultural (AS) and rhizosphere (RS) soil clone libraries, coded as ‘CA’, ‘CB’, ‘CC’, and ‘CD’ respectively, and closely related *cbbM* gene sequences from known cultured representatives and environmental clones. The scale bar indicates 0.5 substitutions per site. The *cbbM* gene sequence of *Methanohalophilus mahii* was used as outgroup for tree calculations.

The composite phylogenetic trees were generated from representative phylotypes of *cbbM*, *apsA* and *soxB* functional genes, together with closely related reference nucleotide sequences retrieved from NCBI. The Maximum Likelihood phylogeny of *cbbM* gene sequences allocated the phylotypes into site-specific clusters that did not show any affiliation to cultured representatives and can be explained by Cluster A, B and C (**Figure 1**). In addition to these clusters, clades 1, 2 and 6 showed a SS1 site-specific distribution and were related to cultured representatives such as *Rhodovulum sulfidophilum* and *Rhodopseudomonas palustris* (Figure 1a). Cluster 8 mostly showed a SS2 site-specific distribution, but also contained one phylotype at the SS1 site and showed a close affiliation to *Thiohalomonas nitratireducens* and *Thiohalomonas denitrificans*.

**Figure 2:**
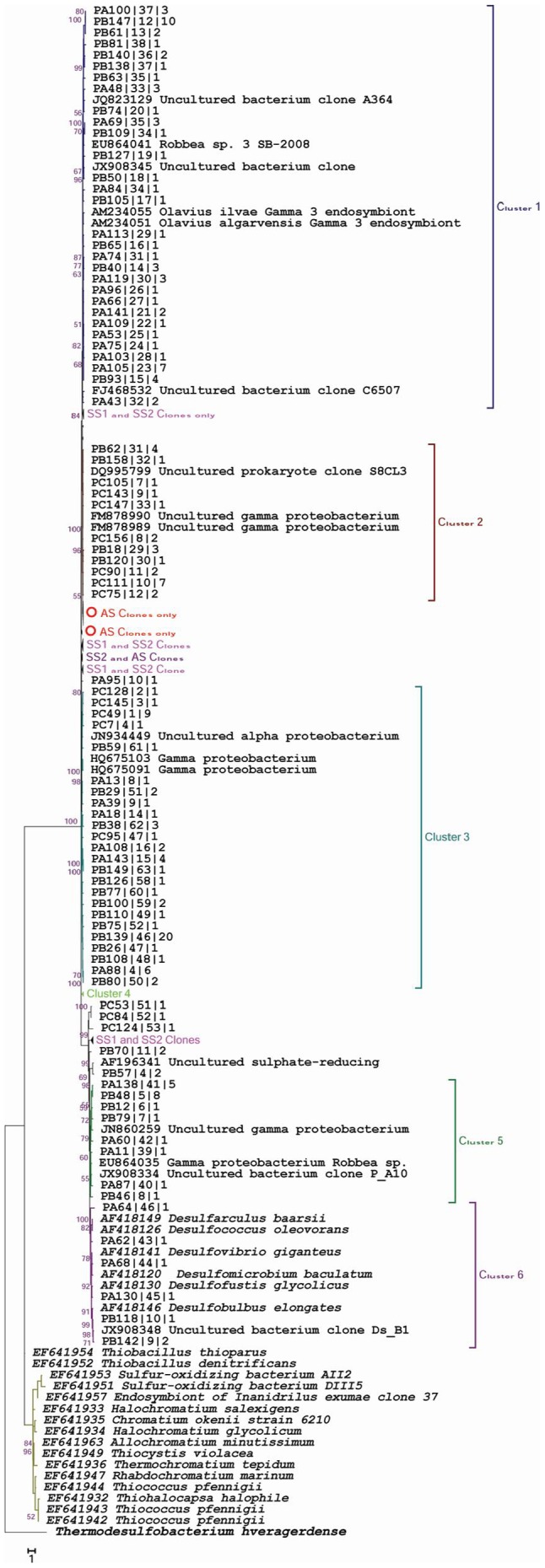
Phylogenetic analysis of the *apsA* gene clones. The final dataset of 215 nucleotide sequences include sequences from low saline soil (SS1), high saline soil (SS2), agricultural soil (AS) clone libraries, coded as ‘PA’, ‘PB’ ‘PC’ respectively, and closely related *apsA*-gene sequences from known cultured representatives and environmental clones. 500 bootstrap analyses were performed and percentages are shown at nodes. The scale bar indicates 1.0 substitutions per site. The *apsA* gene sequence of *Thermodesulfobacterium hveragerdense* was used as outgroup for tree calculations.

The *apsA* nucleotide sequences of all three clone libraries revealed site-specific partitioning and could be distributed into six major clusters (**Figure 2**). The majority of phylotypes (60%) grouped together with uncultured bacteria and showed no affiliation to any cultured representatives, and therefore, could be considered as novel lineages. Cluster 6 consisted of sequences that grouped together with sulphur-reducing bacterial groups such as *Desulfofustis glycolicus*, *Desulfomicrobium baculatum* and *Desulfococcus oleovorans*.

**Figure 3:**
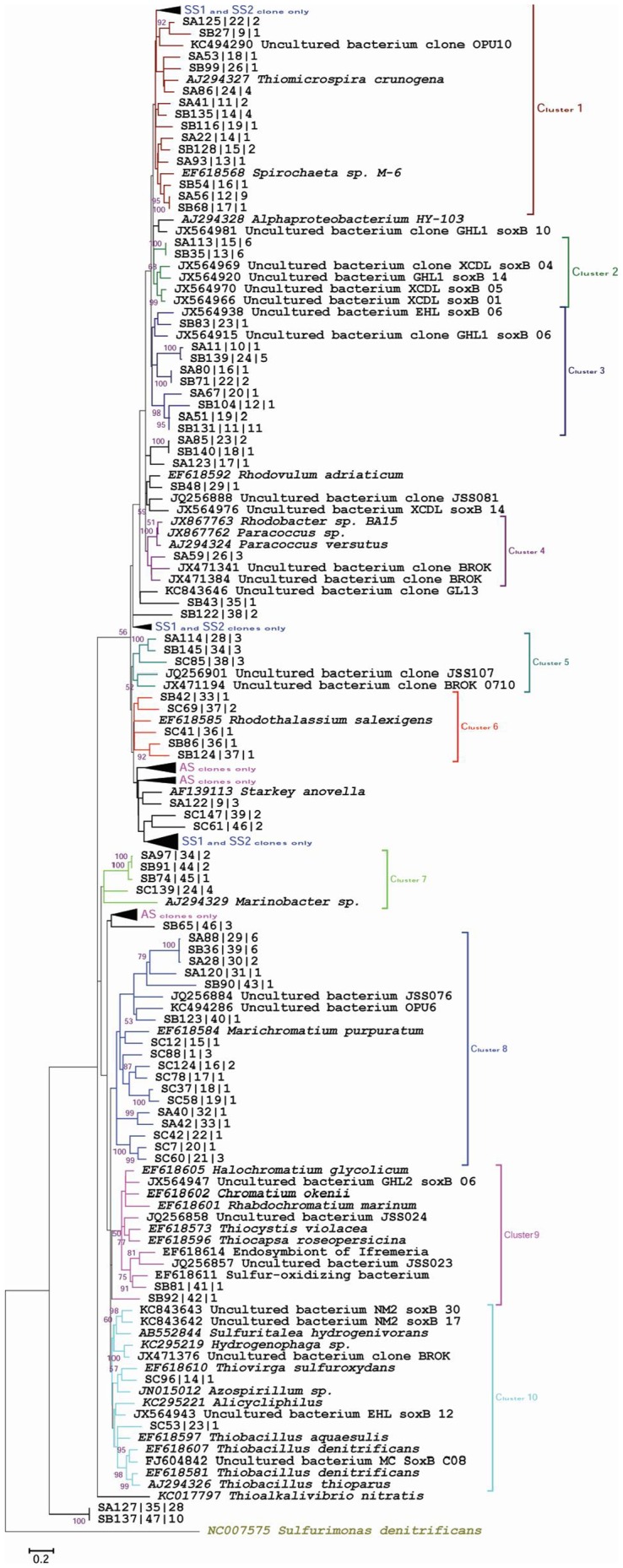
Phylogenetic analysis of the *soxB* gene clones. The final dataset of 186 nucleotide sequences include sequences from low saline (SS1), high saline (SS2), agricultural (AS) soil clone libraries coded as ‘SA’, ‘SB’ ‘SC’ respectively, and closely related *soxB* gene sequences from known cultured representatives and environmental clones. 500 bootstrap analyses were performed and percentages are shown at nodes. The scale bar indicates 0.2 substitutions per site. The *apsA* gene sequence of *Sulfurimonas denitrificans* was used as outgroup for tree calculations.

The *soxB* nucleotide sequences grouped into ten major clusters in the phylogenetic tree and showed affiliation to *soxB* genes from different cultured references (**Figure 3**). The majority of sequences (40% of clones) also formed site-specific clusters, such as one group of five clusters that only possessed sequences from saline soils and showed no affiliation to cultured representatives. Similarly, three clusters were specific for the AS site (67% clones), and were also not allied to any representative cultured bacteria. Cluster numbers 1, 4 and 9 contained sequences specific to saline soils SS1 and SS2, and showed affiliation to *soxB* genes of different cultured representatives, including *Thiomicrospira crunogena*, *Spirochaeta*, *Rhodobacter*, *Paracoccus* and sulphur-oxidising bacteria. Clusters 5, 6, 7 and 8 comprised sequences from all three libraries and were allied to different cultured representatives, including *Rhodothalassium salexigens*, *Marinobacter* sp. and *Marichromatium purpuratum*. Cluster 10 was specific for the AS site and tightly clustered with *Thiovirga sulfuroxydans*, *Azospirillum* sp. and *Thiobacillus aquaesulis*.

### Diversity indices and community structure based on *cbbM*, *apsA* and *soxB*

The α-diversity indices such as ACE, Chao, the number of observed OTUs and the Shannon and Simpson index were evaluated for all clone libraries. The analysis of these diversity indices indicated the predominance of bacteria harbouring the *cbbM* gene at saline soils (SS1 & SS2), whereas the agricultural soil systems (AS & RS) represented less diverse niches (**Table 1**). The diversity index assessment of the *apsA* and *soxB* gene-clone libraries revealed the dominance of sulphur oxidising bacteria in SS2 and AS habitats and less diversity at SS1. This α-diversity is often represented by a rarefaction curve, which is a plot of the number of observed OTUs as a function of the total number of clones captured. This plot of *cbbM* gene sequences (distance  = 0.05) reached an asymptote in the AS and RS clone libraries, but did not reach saturation in the SS1 & SS2 clone libraries (**Figure S3**). In the *apsA* and *soxB* gene libraries, the rarefaction curves inclined towards an asymptote for SS1, but non-asymptotic curve were produced by the SS2 and AS gene libraries (**Figure S3**).

**Figure 4:**
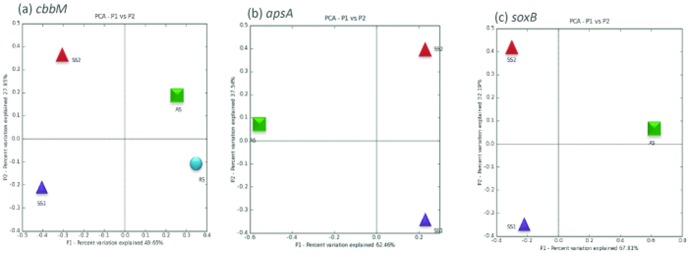
UniFrac PCA of the *cbbM, apsA* and *soxB* gene clone libraries. The ordination plots for the first two dimension to show the relationship between saline soils, agricultural and rhizosphere soil types for (a) *cbbM* (b) *apsA* and (c) *soxB* gene assemblages. The saline soils are represented by purple diamond (SS1) and green circle (SS2), agricultural soil (AS) is represented by circle and rhizosphere soil by blue square. Each axis indicates the fraction of the variance in the data that the axis accounts for.

We employed phylogenetic tree-based comparisons, the UniFrac metric and phylogenetic *P*-test to *cbbM*, *apsA* and *soxB* clone libraries to investigate the β-diversity, which is a measure of the community structure comparison. Weighted UniFrac environmental clustering analysis indicated that the assemblages of bacterial sequences at all four habitats are highly differentiated (UniFrac *P≤0.03*). To determine whether the samples clustered in two dimensional space, PCA was applied to the UniFrac metric. The ordination diagram (**Figure 4a**) of *cbbM* clone libraries revealed that the strongest variation in the data set was between agricultural and saline soils, as these were separated on the first axis of the ordination diagram, which explains the high percentage of total variation (55.51%). For the *apsA* and *soxB* gene clone libraries, the first axis separated agricultural and saline soils, which explains the total community variability (57.78%) among three sample sites (**Figure 4b, c**). The Unifrac analysis revealed the differentiation in community structure and diversity in quite different soil ecosystems which was supported by the *p* significance (*P≤0.03*). The uniqueness at these habitats was further supported by a Venn diagram, which indicated a very low overlap of phylotypes (**Figure S4**). The relative abundance of different phylotypes within the respective clone libraries was depicted by Heatmap (**Figure S5**).

### Abundance of 16S rRNA and functional gene(s) using Real-Time PCR

**Figure 5:**
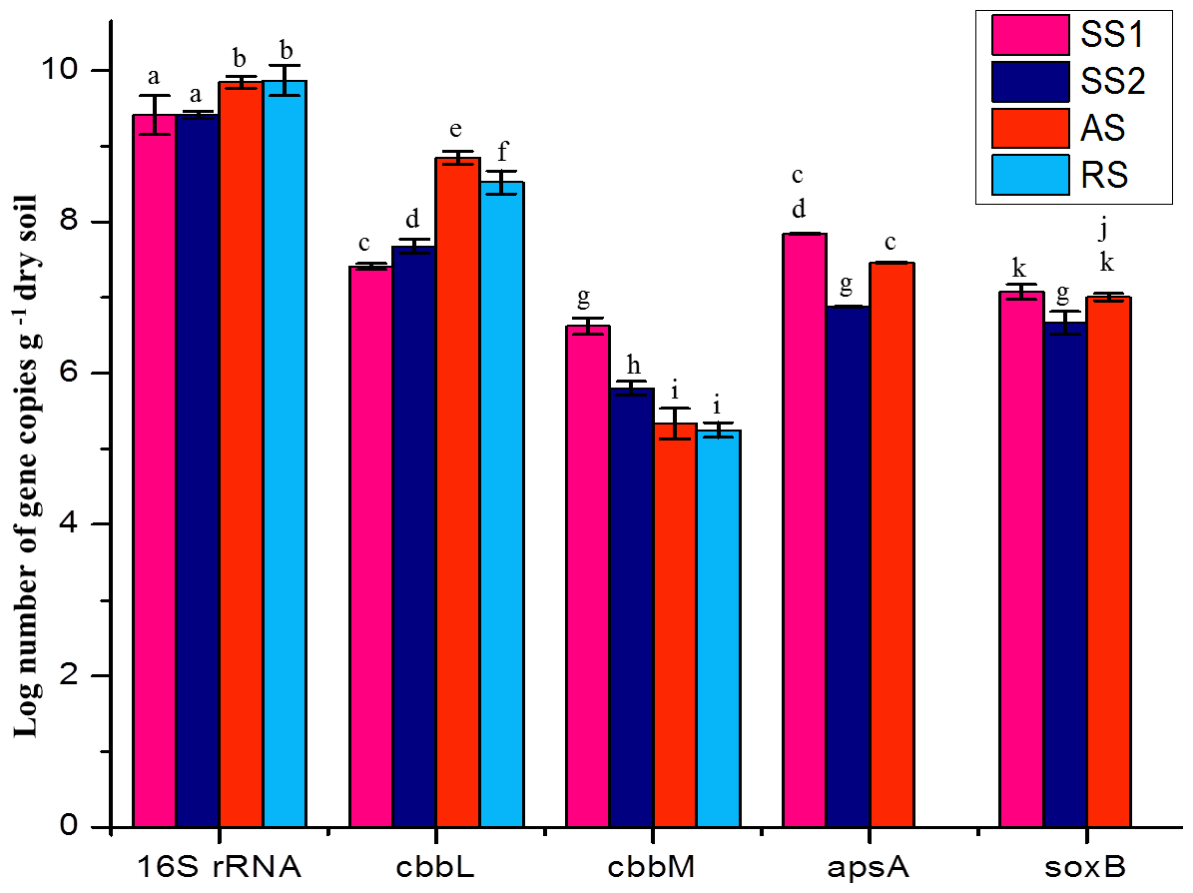
Abundance of the 16S rRNA, *cbbL*, *cbbM*, *apsA* and *soxB* genes in metagenomes of four different soil types determined by qPCR.

The 16S rRNA and functional genes involved in carbon (C) cycling and sulphur (S) are important for sustainable ecosystems. The abundance of these genes (copy number per g soil) was determined in all four soil ecosystems using extracted metagenomes and Real-time PCR. The qPCR results showed a heterogeneous distribution of 16S rRNA gene densities among the four sample sites. The number of gene copies was highest in the rhizosphere and agricultural soil samples, followed by in saline soil SS1 and SS2, with mean values ranging from 7.82×10^9^ to 2.8×10^9^ 16S rRNA copies per g soil (**Table S3**). The abundance of 16S rRNA genes was three-fold higher than that of functional genes. The abundance of *cbbL* genes (per g soil) was significantly higher (*P≤0.05*) in AS than in saline soils (SS1 and SS2). A trend towards an increase in the abundance of *cbbM* gene copies per g soil was observed from rhizospheric soil to saline soil (RS>AS>SS2>SS1) (**Figure 5**). Regarding sulphur cycles, the gene copies of *apsA* and *soxB* genes was significantly higher for the SS1 soil type, followed by in the AS and SS2 types (SS1>AS>SS2) (**Figure 5**).

### Distribution of functional gene OTUs in relation to habitat physicochemical properties

**Figure 6:**
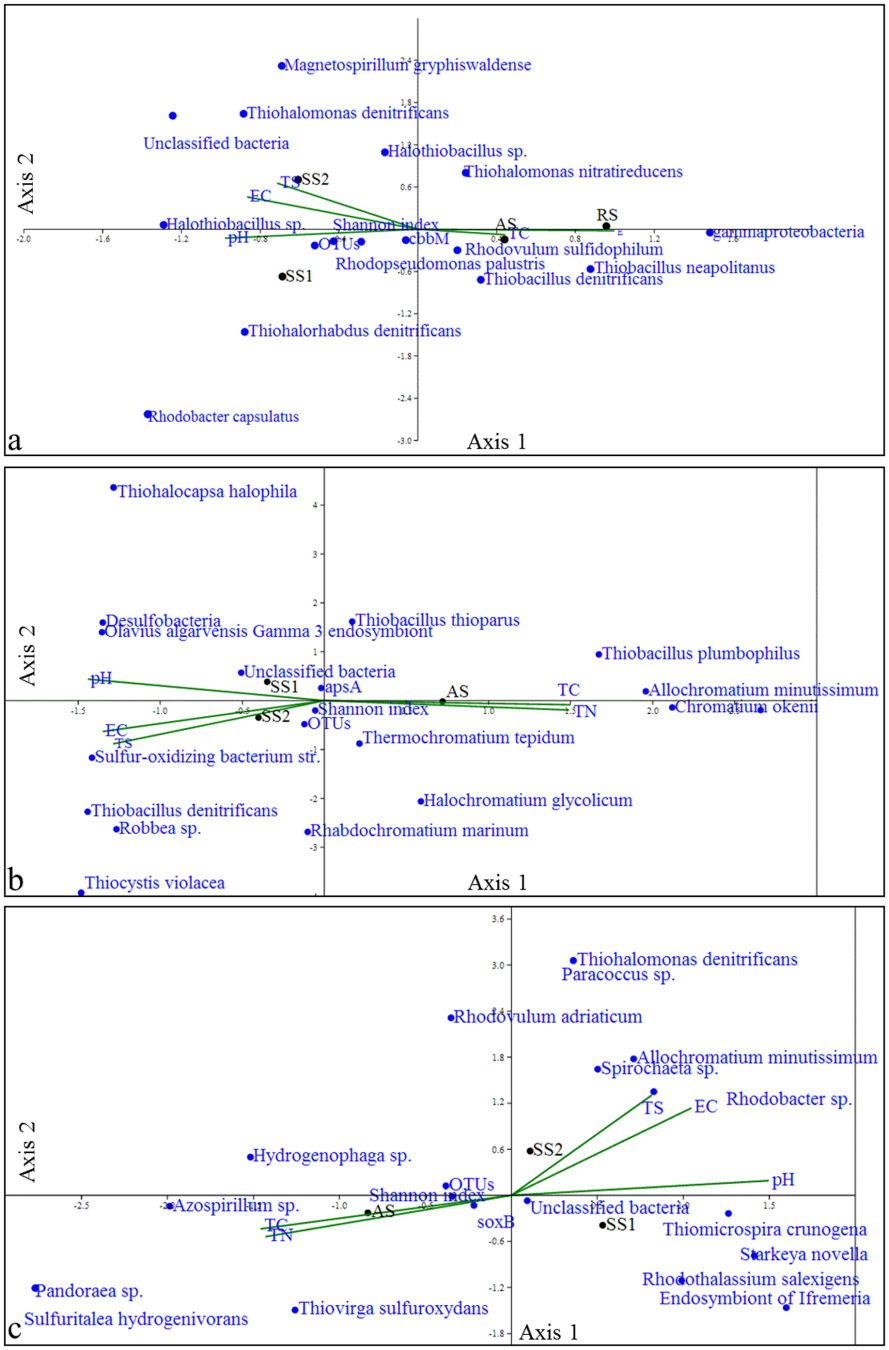
Canonical correspondence analysis. Canonical correspondence analysis (CCA) of nucleotide sequence data sets retrieved from saline soils (SS1 & SS2), agricultural soil (AS) and rhizosphere soil (RS) systems along with selected environmental variables of these four sites. The selected environmental variables include electrical conductivity (EC), pH, total C content (TC), total nitrogen content (TN) and total sulphur content (TS) for four soil types. The CCA ordination diagrams for (a) *cbbM* (b) *apsA* and (c) *soxB* clone libraries.

The distribution of the abundant microbial communities among different sites was analysed using Canonical Correspondence Analysis (CCA) plots (**Figure 6**), which revealed that all the functional genes (*cbbM, apsA* and *soxB*) of microbial communities were marginally significantly (*P≤0.05*) correlated with all the selected environmental variables. The best *P* value that was obtained was 0.06, with an eigen-value of 0.5 following 999 Monte Carlo permutations for any stepwise iteration of the environmental factor and gene sequence data sets. In the *cbbM* clone library, axes 1 and 2 showed variation of 56% and 28.3% respectively (**Figure 6a**). Similarly, in the *apsA* clone library, SS2 strongly positively correlated with EC and TS with a variation for axis 1 of 75.3% (**Figure 6b**). An axis 2 (24.7% of the variance) was correlated with TC and TN, which in turn, was correlated with the ordination of *Allochromatium minutissimum* and *Chromatium okenii*. In the *soxB* clone library, both CCA axes 1 and 2 (58.4%, 41.56% of the variance respectively), were strongly positively correlated with TS, EC and pH and were strongly but negatively correlated with TC and TN (**Figure 6c**). The ordination of *soxB* OTUs designated to taxonomic groups such as *Allochromatium minutissimum*, *Spirochaeta* sp. and *Rhodobacter*, highly correlated with the SS2 site and with TS, EC and pH environmental variables.

## Discussion

This study provides a useful insight into the comparative microbial community structure and the occurrence of potential autotrophic bacterial groups/pathways in coastal saline soils and agricultural/rhizosphere ecosystems. To the best of our knowledge, this study represents the first comprehensive information on functional diversity and the quantification of genes (*cbbM*, *apsA* and *soxB*) associated with autotrophic bacterial biota at these sites using gene-targeted metagenomics.

### *Gammaproteobacteria* as predominant group involved in *cbbM* gene based CO_2_ fixation

The RuBisCO form I, which is based on autotrophic metabolism at these four selected sites [22], [23], revealed that *cbbL*-harbouring autotrophs were exclusively dominated by *Alpha*- and *Betaproteobacteria*. In this study, *cbbM*-based metabolism was envisioned, which is prevalent in microaerobic/anaerobic autotrophs in diverse environments [29]. Similar studies have been performed in variable environments, particularly in aquatic sites such as groundwater, extreme hypersaline habitats and hydrothermal sites [6]–[8], [11], [15], [30]–[31], but have been overlooked in terrestrial habitats. Notably, this gene mostly occurs in organisms that possess the *cbbL* gene [30], which might be highly advantageous to autotrophic bacteria, because the dissimilar kinetic properties of the enzyme allows them to assimilate CO_2_ efficiently in both aerobic and anaerobic conditions [30].

In all four *cbbM* libraries, we recovered a number of gene clones that were related to *Gammaproteobacteria*, which are hypothesised to oxidise sulphur in marine sediments, based on their 16S rRNA phylogenetic relationship to other uncultured sulphur-oxidising bacteria [14]. The large number of these most consistently occurring phylotypes were ascribed to different carbon fixing genera that are reported to be associated with biogeochemical transformations in extreme environments [29]. Clones related to *Rhodopseudomonas palustris*, which is a metabolically versatile bacterium capable of anoxygenic photosynthesis under anaerobic conditions using a variety of carbon sources [32], were observed in SS1, SS2 and AS clone libraries. *Thiohalorhabdus denitrificans* is an extremely halophilic, sulphur-oxidising, deep-lineage *Gammaproteobacteria* from hypersaline habitats [33]. The nine clones from the AS library showed affiliation to *Thiobacillus denitrificans*, which shows obligate autotrophic metabolism and obtains energy for CO_2_ fixation by combining the oxidation of inorganic sulphur compounds with denitrification [34]. This bacterium has a wide distribution and plays a major role in biogeochemical cycles on a global scale, especially linking sulphur and nitrogen cycles. *Rhodovulum sulfidophilum*-related sequences were exclusively dominant in the AS library, but these microbes are commonly reported from marine and high salt environments and can utilise both reduced sulphur compounds; sulphide and thiosulphate [35]. The presence of sequences belonging to *Gammaproteobacteria* in the 16S rRNA gene library in our previous study [23] as well as in the *cbbM* gene libraries, demonstrates its importance in saline soil ecosystems, which suggests that the *Gammaproteobacteria*-related RuBisCO might contribute to primary production in such environments [14]. The results of 16S rRNA, *cbbL* and *cbbM* clone libraries collectively indicate that sulphur might be the governing element in supporting chemolithoautotrophic communities in saline ecosystems. The majority of the clone sequences from saline (75%) and agricultural soil (72%) clone libraries did not cluster with cultured representatives in the molecular phylogenetic tree (**Figure 1**) and thus could be considered to be novel genes. The identification of these novel genes will contribute to knowledge about the genetic pool of these genes at these habitats, as well as to the databases.

### Gammaproteobacteria and Alphaproteobacteria as the dominant sulphur oxidisers

Because sulphur metabolism occurs via multiple oxidation pathways, this study targeted the key genes (*apsA* & *soxB*) of two important sulphur oxidation pathways [12]. The study revealed the lack of *soxB* gene amplification in the RS metagenome (**Table S1** and **Figure S2**), although this gene has been reported in various cultured rhizobacteria isolated from crop plants [36]. This might be due to the absence or inadequate representation of sulphur-oxidising bacteria in rhizosphere soil and primer bias cannot be completely ruled out. The saline soils were dominated by phylotypes affiliated to *Alpha*- and *Gammaproteobacteria*, such as *Rhodothalassium salexigens*, *Thiomicrosporra crunogena*, *Paracoccus*, *Spirochaeta* and *Rhodovillum adriaticum*; *however, Betaproteobacteria*-related phylotypes dominated the AS site and were putative sulphide oxidisers, which agrees with our previous report on the AS 16S rRNA clone library [24]. The *soxB* gene has been detected in anaerobic anoxygenic phototrophic members of the *Alphaproteobacteria*, such as *Rhodothalassium*, *Rhodospirillum* and *Rhodovulum* [16]. Notably, *soxB* phylotypes related to those of *Rhodothalassium salexigens* (15 clones) and *Rhodovulum adriaticum* (17 clones) were most dominant putative sulphide oxidisers in SS1 and SS2 clone libraries, respectively. The *Thiomicrospira crunogena*- and *Paracoccus*- like phylotypes were the other dominant group in the SS1 clone library, which are obligately chemolithoautotrophic sulphur-oxidising *Gammaproteobacteria* and *Alphaproteobacteria*, respectively, and were restricted to saline soil ecosystems only. The *Paracoccus*-like phylotypes were also retrieved from saline soil *cbbL* clone libraries, as was observed in our previous study [23], reflecting their active role in saline habitats. *Thiomicrospira* sp. has been demonstrated to oxidise thiosulphate to sulphate using the *Paracoccus pantotrophus* homologous *sox* cluster, despite the absence of *aps* genes [37]. Representatives of the *Thiomicrospira* group behave as obligate autotrophs, which use form I RuBisCO (green-like) and form II RuBisCO as the key enzymes for inorganic carbon assimilation [38]. It is notable that the green-like *cbbL* gene could only be amplified from the SS2 site in our previous study [23]. This, together with the results here, suggests that the *Thiomicrospira* group might play an active role at this site. These dominant, recognised genera, can oxidise sulphur, and can thus play a prominent role in biogeochemical sulphur and carbon cycling. The AS clone library was dominated by *Gammaproteobacteria*, *Betaproteobacteria*-related phylotypes, including *Marichromatium purpuratum* and *Thiobacillus*. *Thiobacilli* spp. are aerobic and anaerobic sulphide-oxidising bacteria that possess *sox* genes [34] and were here restricted to agriculture soil.

Previously, the *Chloroflexi* group was observed in saline-soil sites [23], but in the present study, *soxB* gene libraries showed no affiliation to members of the *Chloroflexi* group. This agrees with the report that the primers used for *soxB* gene amplification did not amplify the *soxB* genes of *Epsilonproteobacteria* and *Chloroflexi* [16]. Moreover, *soxB* genes are not reported in members of the *Chloroflexi* group, and *Epsilonproteobacteria* were not represented in our 16S rRNA library. The *soxB* genes identified from all the analysed libraries led us to conjecture about the global distribution of these genes. This widespread occurrence could be attributed to horizontal gene transfer, as revealed by *soxB*-based phylogenies [16], [39]. In the *soxB* clone library, *Betaproteobacteria* predominantly represented the AS site, which agrees with data from the previous study, where *Betaproteobacteria* was predominant in freshwater/low-salinity environments [40] and also reinforced previous results with the 16S rRNA clone library, in which the *Betaproteobacteria* group was highly abundant at this site.

The composite molecular phylogeny envisages that 40% of saline and 60% of agricultural soil clones were not affiliated to any cultured representatives harbouring *soxB*, which indicated a high unprecedented novel diversity (**Figure 3**). Numerous phylotypes showed a very clear connection to potential sulphur oxidisers such as *Thiomicrospira crunogena, Rhodothalassium* and *Paracoccus*, suggesting the potential for sulphur-based metabolism for energy generation at these sites, in addition to Calvin cycle.

The key enzyme aps reductase, performs sulphur metabolism in both reductive and oxidative pathways [41] and the *apsA* gene is considered to be a remarkable tool for investigating sulphur-oxidising prokaryotes. The majority of the recovered sequences in all three *apsA* clone libraries grouped with *Gammaproteobacteria* (SS1, SS2, AS; 29, 70, 81 clones), many of which were affiliated with potential sulphur oxidisers. Most of these AS *Gammaproteobacteria* SOPs were anaerobic anoxygenic phototrophs (*Chromatiaceae*), indicating that aerobic or microaerobic conditions prevailed at this site. Only few saline soil *apsA* gene clone sequences were related to those from these anaerobic anoxygenic phototrophs. The *apsA* saline-soil clone libraries most frequently contained phylotypes (SS1 and SS2-76 and 53 clones), which were related to those of uncultured clones and could be considered novel genes from yet un-described sulphur oxidisers. The results illustrate the under-exploration of the sulphur-oxidising microflora at these sites, which could be a pool for potential novel strains involved in sulphur metabolism. Many of the phylotypes were related to the symbiotic SOP-like *Olavius ilvae*, *O. algarvensis* and *Robbea* Gamma 3 endosymbionts, which are gutless oligochaete worms from Mediterranean seagrass sediments [42]. These co-occurring symbionts are sulphur-oxidising and sulphate-reducing bacteria and can assimilate CO_2_ autotrophically, which enables them to provide multiple sources of nutrition to the particular host [43]–[44]. The absence of *apsA* gene amplification in the RS metagenome corresponded to *soxB* gene, further confirmed the lack of sulphur-oxidising representatives in this habitat. The numerically largest group of *apsA* phylotypes from the AS library were related to *Chromatium okenii* (39 clones) and *Allochromatium minutissimum* (33 clones). The ecological versatility of *Allochromatium* strains causes them to inhabit numerous habitats, such as stagnant freshwater ditches, ponds, lakes, sewage lagoons, estuaries and salt marshes. Five phylotypes from the SS2 library were related to *Allochromatium vinosum*, which can switch from anaerobic to aerobic sulphur oxidation. This genus was formerly referred to as *Chromatium vinosum*, a representative of the *Chromatiaceae* and possesses two sets of divergent genes that encode the green-like RuBisCO enzyme [45]. The saline-soil libraries (SS1 & SS2) possessed limited phylotypes that represented *Deltaproteobacteria* members such as *Desulfarculus baarsii* and a few members of *Alpha*- and *Betaproteobacteria*. The *Beta*- and *Gammaproteobacteria* were the most frequent sulphur oxidisers, whereas sulphur-reducing bacteria most frequently belonged to *Deltaproteobacteria*, as reported in previous studies [46]. The composite phylogeny of *apsA* genes revealed that the majority of clone sequences (60%) did not show affiliation to well-recognized sulphur-oxidising bacteria, prompting us to presume their novelty and uniqueness (**Figure 2**).

### Gene abundance

Bacterial 16S rRNA gene copy numbers ranged from 2.6×10^9^ to 7.8×10^9^ per g of dry soil; SS2 had the fewest copies, followed by SS1, AS and RS (**Table S3**), which is comparable to the numbers measured in other soil ecosystems using real-time PCR [18], [47]–[48]. Previously, about 1×10^11^ 16S rRNA copies were reported from paddy soils [11]. The 16S rRNA gene copy number was two to four orders of magnitude higher than that of all studied functional genes. The precise estimation of copy number was difficult, as the number of 16S rRNA gene copies per bacterial cell ranged from 1–15 [49]. The results showed that the *cbbL* gene copy numbers significantly (*P≤0.05*) outnumbered *cbbM*. This result agrees with results from the present clone library analysis, as well as with research performed in paddy soils [11]. The copy number of the *cbbL* gene was comparable to that in the study on paddy soil [50]–[51] and somewhat higher than that reported in other bulk soil niches [52]–[53]. Moreover, because the number of copies of the *cbb* operon in bacteria varies [38], the number of copies calculated could not be precisely defined.

The abundance of *soxB* has not been established in terrestrial ecosystems, but the gene has been reported from extreme water systems such as Qinghai-Tibetan Lakes; hypersaline lakes [19] and the copy number in sediment was comparable with that in the present data (**Table S3**). To the best of our knowledge, there has only been one report on *apsA* gene abundance in soil ecosystems to date, [18] and the copy number of the *apsA* gene observed in saline–alkaline soil contrasted with that detected in this study. The differential abundance of these metabolic marker genes can be attributed to phylogenetically diverse copies of functional genes such as *apsA,* which can be present in a single species [17]. The abundance of *apsA* and *soxB* genes was comparable at each site, which corresponded with the results observed for *apsA* and *dsrB* [54], where the copy number for both genes was almost equal at all sites.

## Conclusions

In this study, we have reported the comparative occurrence of functional genes involved in different modes of autotrophy among four contrasting terrestrial habitats and envisaged their composite phylogeny for the first time. The collective data obtained revealed that *Gammaproteobacteria* and *Alphaproteobacteria* were the abundant groups and represented major fractions of the sequences of the *cbbM*, *apsA* and *soxB* clone libraries from saline-soil ecosystems. Thus, we hypothesise from the results, that novel *Gammaproteobacteria* sulphur-oxidisers might play a prominent role in primary production and might be heavily involved in the cycling of carbon and sulphur compounds, principally at saline-soil sites. This study requires other collaborative approaches to confirm the hypothesis concerning metabolic functioning in these ecosystems, such as novel bacterial isolation and stable isotope probing. Nevertheless, this study detected a pattern in the distribution of functional microbial guilds across these sites, which is a prerequisite for the ecological understanding and ascertaining of autotrophic physiology at these sites.

## Supporting Information

Figure S1 Distribution of functional microbial groups across four different soil habitats.
(PDF)

Figure S2 **PCR amplification of targeted functional genes.** PCR amplification of *cbbM, apsA, aclB* and *soxB*, using different soil DNA as template following their respective gene specific primers and PCR conditions (Table S1). M: Marker (100 bp DNA ladder); SS1, SS2, AS, and RS: Soil Samples; PC: Positive control.
(PDF)

Figure S3 **Rarefaction curves for targeted functional genes clone library.** Rarefaction curves for (a) *cbbM* (b) *apsA* and (c) *soxB* gene clone libraries at 0.05 cut-off. Bacterial richness in SS1, SS2, AS and RS soils is indicated by slopes of the rarefaction curves.
(PDF)

Figure S4 **Venn diagrams for targeted functional genes.** Venn diagrams representing the observed overlap of OTUs for (a) *cbbM*, (b) *apsA* and (c) *soxB* gene libraries (distance  = 0.05). Venn diagrams show overall overlap of representative genera between soils. The values in the diagram represent the number of genera that were taxonomically classified.
(PDF)

Figure S5 **Heat map analysis.** Heat map showing abundance of OTUs in (a) *cbbM*, (b) *apsA* and (c) *soxB* gene clone libraries (distance  = 0.05). Each row in the heatmap represents a different OTU and the colour of the OTU in each group scaled between black and red according to the relative abundance of that OTU within the group.
(PDF)

Table S1 **Primer sets used for the study of microbial community structure and gene abundance.**
(PDF)

Table S2 **Physicochemical characteristics of soil samples.**
(PDF)

Table S3 **Copy no. of gene(s) determined by qPCR in soil metagenomes.**
(PDF)
