# Supplementary figures and images for: Unravelling the Carbon and Sulphur Metabolism in Coastal Soil Ecosystems Using Comparative Cultivation-Independent Genome-Level Characterisation of Microbial Communities
Source: PLoS One. 2014 Sep 16;9(9):e107025. doi: 10.1371/journal.pone.0107025 (PMC4167329; doi:10.1371/journal.pone.0107025)

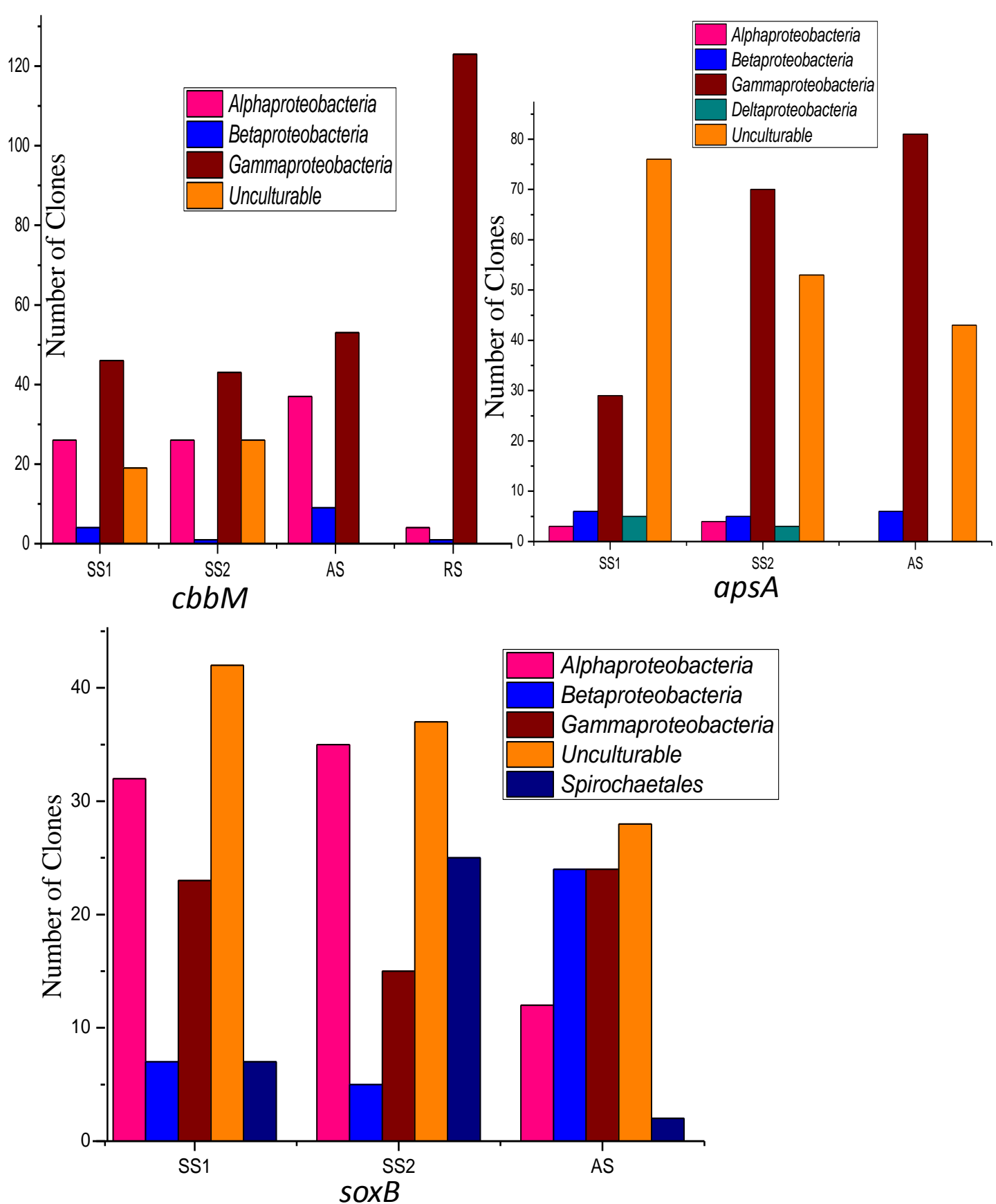

**Figure S1: Distribution of functional microbial groups across four different soil habitats**

Supplement: Figure S1 — Distribution of functional microbial groups across four different soil habitats. (PDF) [file pone.0107025.s001.pdf]
