# Supplementary material for: Unravelling the Carbon and Sulphur Metabolism in Coastal Soil Ecosystems Using Comparative Cultivation-Independent Genome-Level Characterisation of Microbial Communities
Source: PLoS One. 2014 Sep 16;9(9):e107025. doi: 10.1371/journal.pone.0107025 (PMC4167329; doi:10.1371/journal.pone.0107025)
Supplement: Figure S2 — PCR amplification of targeted functional genes. PCR amplification of cbbM, apsA, aclB and soxB, using different soil DNA as template following their respective gene specific primers and PCR conditions (Table S1). M: Marker (100 bp DNA ladder); SS1, SS2, AS, and RS: Soil Samples; PC: Positive control. (PDF) [file pone.0107025.s002.pdf]

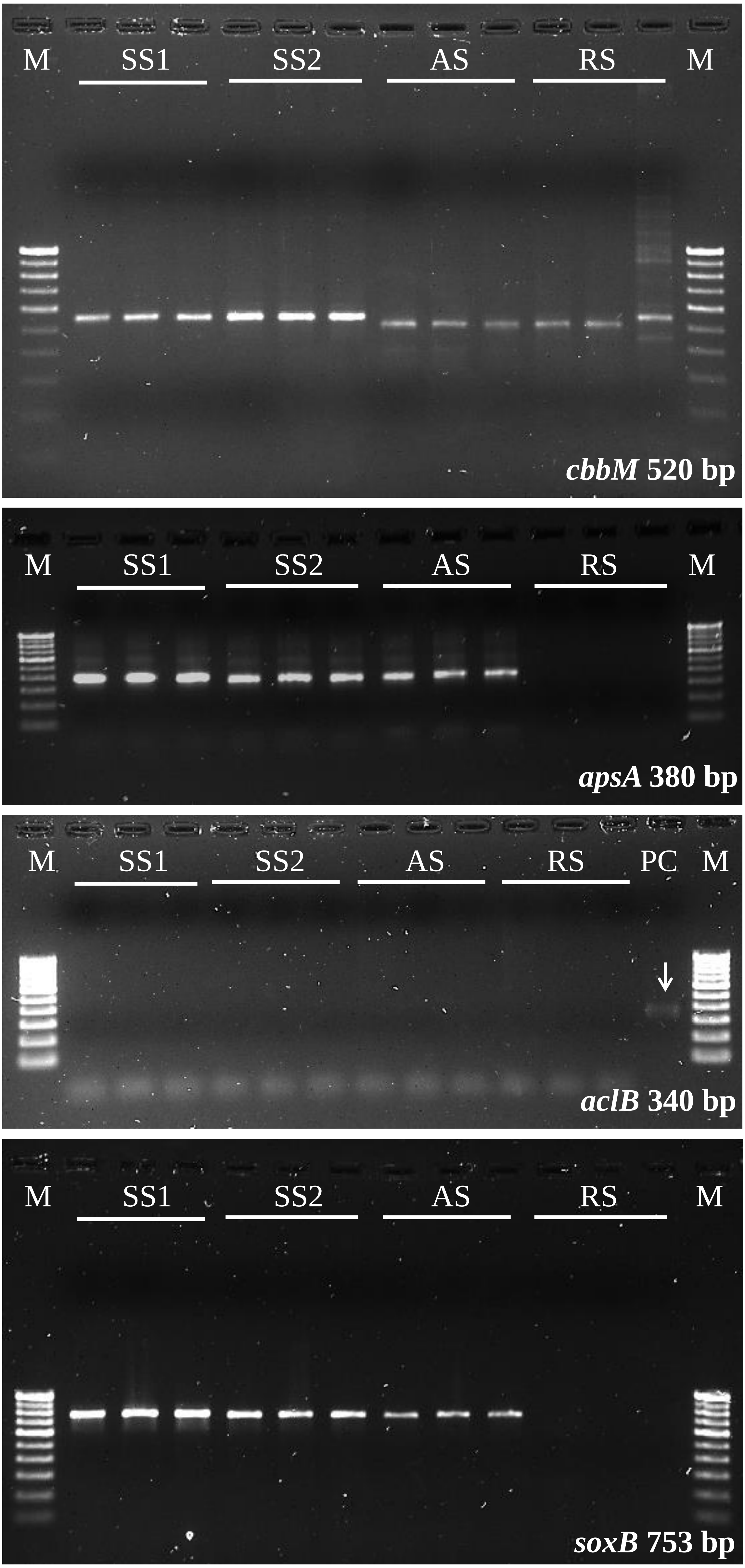

Figure S2: PCR amplification of targeted functional genes. PCR amplification of *cbbM*, *apsA*, *aclB* and *soxB*, using different soil DNA as template following their respective gene specific primers and PCR conditions (Table S1). M: Marker (100 bp DNA ladder); SS1, SS2, AS, and RS: Soil Samples; PC: Positive control
