# Supplementary material for: Unravelling the Carbon and Sulphur Metabolism in Coastal Soil Ecosystems Using Comparative Cultivation-Independent Genome-Level Characterisation of Microbial Communities
Source: PLoS One. 2014 Sep 16;9(9):e107025. doi: 10.1371/journal.pone.0107025 (PMC4167329; doi:10.1371/journal.pone.0107025)
Supplement: Figure S3 — Rarefaction curves for targeted functional genes clone library. Rarefaction curves for (a) cbbM (b) apsA and (c) soxB gene clone libraries at 0.05 cut-off. Bacterial richness in SS1, SS2, AS and RS soils is indicated by slopes of the rarefaction curves. (PDF) [file pone.0107025.s003.pdf]

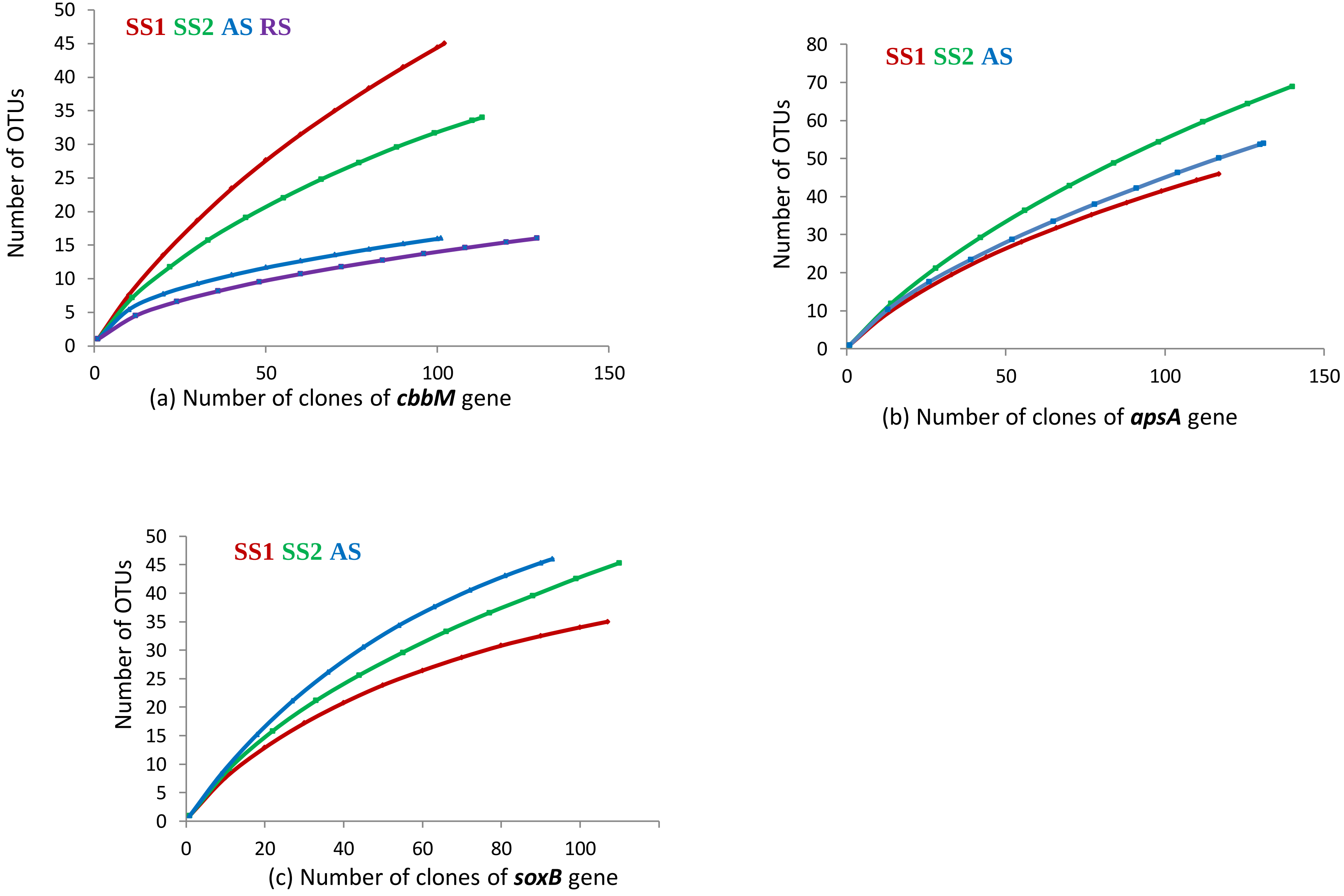


**Figure S3: Rarefaction curves for targeted functional genes clone library.** Rarefaction curves for (a) *cbbM* (b) *apsA* and (c) *soxB* gene clone libraries at 0.05 cut-off. Bacterial richness in SS1, SS2, AS and RS soils is indicated by slopes of the rarefaction curves.
