# Supplementary material for: Unravelling the Carbon and Sulphur Metabolism in Coastal Soil Ecosystems Using Comparative Cultivation-Independent Genome-Level Characterisation of Microbial Communities
Source: PLoS One. 2014 Sep 16;9(9):e107025. doi: 10.1371/journal.pone.0107025 (PMC4167329; doi:10.1371/journal.pone.0107025)
Supplement: Figure S4 — Venn diagrams for targeted functional genes. Venn diagrams representing the observed overlap of OTUs for (a) cbbM, (b) apsA and (c) soxB gene libraries (distance = 0.05). Venn diagrams show overall overlap of representative genera between soils. The values in the diagram represent the number of genera that were taxonomically classified. (PDF) [file pone.0107025.s004.pdf]

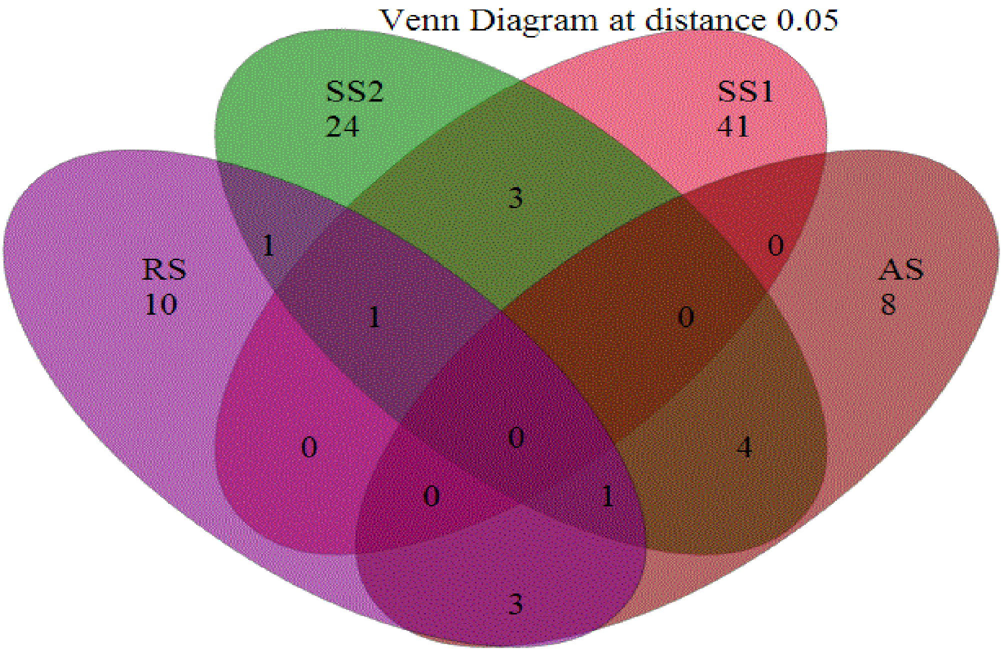

(a) *cbbM*

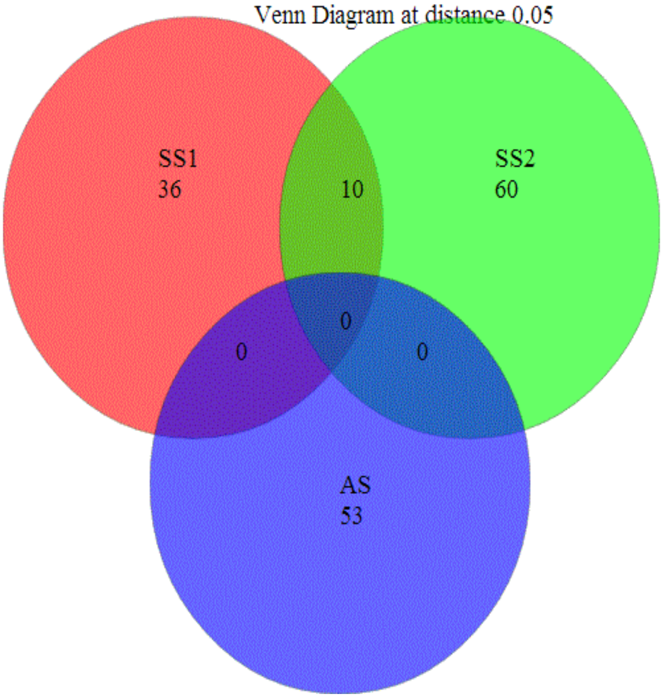

(b) *apsA*

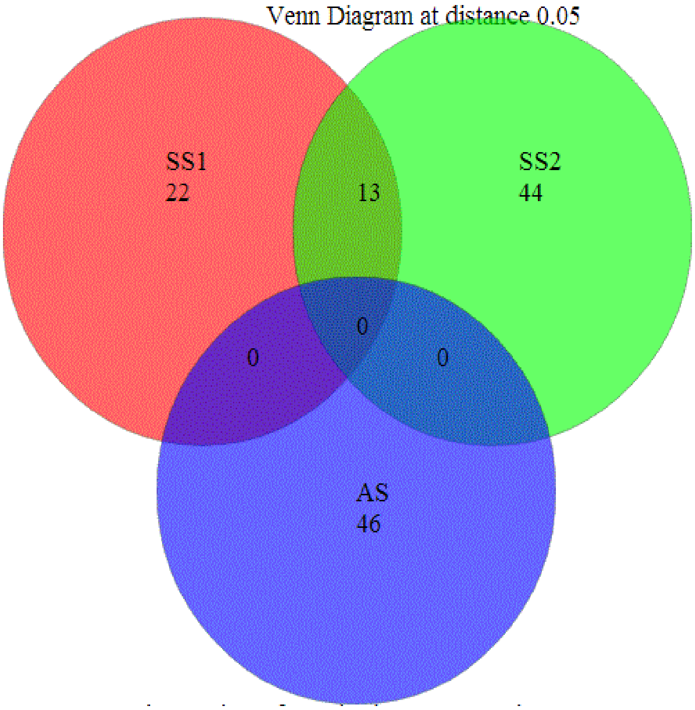

(c) *soxB*

Figure S4: Venn diagrams representing the observed overlap of OTUs for (a) *cbbM*, (b) *apsA* and (c) *soxB* gene libraries (distance = 0.05).Venn diagrams show overall overlap of representative genera between soils. The values in the diagram represent the number of genera that were taxonomically classified.
