# Supplementary material for: Unravelling the Carbon and Sulphur Metabolism in Coastal Soil Ecosystems Using Comparative Cultivation-Independent Genome-Level Characterisation of Microbial Communities
Source: PLoS One. 2014 Sep 16;9(9):e107025. doi: 10.1371/journal.pone.0107025 (PMC4167329; doi:10.1371/journal.pone.0107025)
Supplement: Figure S5 — Heat map analysis. Heat map showing abundance of OTUs in (a) cbbM, (b) apsA and (c) soxB gene clone libraries (distance = 0.05). Each row in the heatmap represents a different OTU and the colour of the OTU in each group scaled between black and red according to the relative abundance of that OTU within the group. (PDF) [file pone.0107025.s005.pdf]

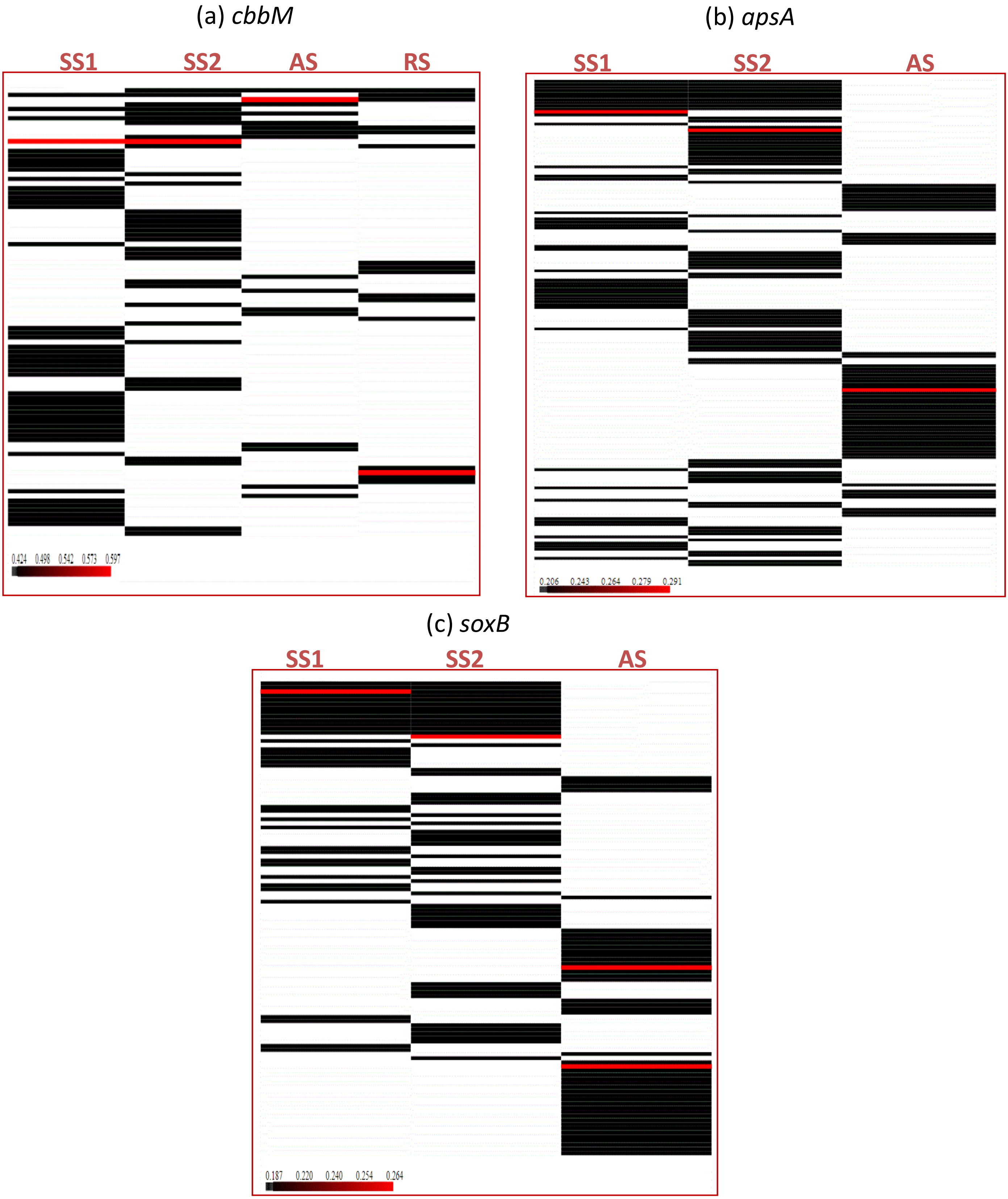

Figure S5: Heat map showing abundance of OTUs in (a)*cbbM*, (b) *apsA* and(c) *soxB* gene clone libraries (distance = 0.05). Each row in the heatmap represents a different OTU and the colour of the OTU in each group scaled between black and red according to the relative abundance of that OTU within the group.
