# Supplementary material for: Unravelling the Carbon and Sulphur Metabolism in Coastal Soil Ecosystems Using Comparative Cultivation-Independent Genome-Level Characterisation of Microbial Communities
Source: PLoS One. 2014 Sep 16;9(9):e107025. doi: 10.1371/journal.pone.0107025 (PMC4167329; doi:10.1371/journal.pone.0107025)
Supplement: Table S1 — Primer sets used for the study of microbial community structure and gene abundance. (PDF) [file pone.0107025.s006.pdf]

Table S1 Primer sets used for the study of microbial community structure and gene abundance

Primer	Primer sequence(5′-3′)	Targeted key enzyme	PCR conditions*	Reference	Soil Samples			
					SS1	SS2	AS	RS
cbbM-f<br>*cbbM*-r	GGCACCATCATCAAGCCCAAG<br>TCTTGCCGTAGCCCATGGTGC	Form II RuBisCO	94 °C/3 min; 25 cycles of 94 °C/30 s 62 °C/30 s 72 °C/45 s; 10 cycles of 94 °C/20 s, 52 °C/20 s 72 °C/45 s; 72°C/ 7 min.	Alfreider *et al.*, 2003	+	+	+	+
apsAF<br>*apsAR*	TGGCAGATMATGATYMACGG<br>GGGCCGTAACCGTCCTTGAA	Adenosine phosphosulfate	95°C/ 5 min; 35 cycles of 95°C/ 1 min 56°C/ 2 min 72°C 2 min;72°C/ 7 min.	Friedrich, 2002	+	+	+	-
aclB-F1<br>*aclB*-R	TGGACMATGGTDGCYGGKGGT<br>ATAGTTKGGSCCACCTCTTC	ATP citrate lyase	94°C for 3 min, 37°C for 2 min, and 72°C for 3 min (2 cycles), followed by 35 cycles of 94°C for 30 s, 54°C for 30 s, and 72°C for 1 min.	Campbell *et al.*, 2003	-	-	-	-
soxBF<br>*soxBR*	ATCGGNCARGCNTTYCCNTA<br>CATGTCNCCNCCRTGYTG	Sulfate thiohydrolase	94 °C/3 min; 10 cycles of 94 °C/30 s 55 °C/40 s 72 °C/30 s; 25 cycles of 94 °C/30 s, 47 °C/40 s 72 °C/30 s; 72°C/ 7 min.	Meyer *et al.*, 2007	+	+	+	-

(+): Positive PCR amplification, (−): no PCR amplification

*Used for normal PCR for the construction of gene libraries however, for quantitative real-time PCR (qPCR) conditions were: 95°C/5 min, 35 cycles of 95°C/ 30 sec, followed by 60°C/ 30 sec; 56°C/ 30 sec and 55°C/ 30 sec for *cbbM*, *apsA* and *soxB* genes respectively.
