# Supplementary material for: Unravelling the Carbon and Sulphur Metabolism in Coastal Soil Ecosystems Using Comparative Cultivation-Independent Genome-Level Characterisation of Microbial Communities
Source: PLoS One. 2014 Sep 16;9(9):e107025. doi: 10.1371/journal.pone.0107025 (PMC4167329; doi:10.1371/journal.pone.0107025)
Supplement: Table S2 — Physicochemical characteristics of soil samples. (PDF) [file pone.0107025.s007.pdf]

Table S2 Physicochemical characteristics of soil samples

Site	EC (dSm^{-1})	pH	TC (%)	TIC (%)	TOC (%)	TN (%)	TS (%)
SS1	3.8±0.07	8.3±0.11	1.27±0.08	0.02±0.08	0.44±0.06	0.09±0.005	0.11±0.01
SS2	7.1±0.10	8.0±0.14	1.38±0.06	0.78±0.06	0.61±0.05	0.09±0.006	0.28±0.02
AS	0.12±0.02	7.09±0.17	2.65±0.19	1.6±0.19	1.04±0.15	0.14±0.006	0.016±0.005
RS	0.13±0.04	6.9±0.08	1.47±0.06	1.01±0.06	0.46±0.7	0.17±0.01	0.05±0.001

Values are shown as Mean ± S.D. of triplicate (n=3) data sets
