# Supplementary material for: Unravelling the Carbon and Sulphur Metabolism in Coastal Soil Ecosystems Using Comparative Cultivation-Independent Genome-Level Characterisation of Microbial Communities
Source: PLoS One. 2014 Sep 16;9(9):e107025. doi: 10.1371/journal.pone.0107025 (PMC4167329; doi:10.1371/journal.pone.0107025)
Supplement: Table S3 — Copy no. of gene(s) determined by qPCR in soil metagenomes. (PDF) [file pone.0107025.s008.pdf]

Table S3 Copy no. of gene(s) determined by qPCR in soil metagenomes

Copy no.	16S rRNA	*cbbL*	*cbbM*	*apsA*	*soxB*
SS1	$(2.8\pm0.25)x10^{9}$	$(2.33\pm0.038)x10^{7}$	$(4.00\pm0.10)x10^{6}$	$(7.0\pm0.008)x10^{7}$	$(7.07\pm0.09)x10^{7}$
SS2	$(2.62\pm0.04)x10^{9}$	$(5.0\pm0.09)x10^{7}$	$(6.67\pm0.08)x10^{5}$	$(7.3\pm0.002)x10^{6}$	$(6.66\pm0.15)x10^{6}$
AS	$(6.37\pm0.07)x10^{9}$	$(7.33\pm0.08)x10^{8}$	$(2.0\pm0.19)x10^{5}$	$(3.0\pm0.013)x10^{7}$	$(7.00\pm0.045)x10^{7}$
RS	$(7.82\pm0.20)x10^{9}$	$(3.6\pm0.15)x10^{8}$	$(1.67\pm0.09)x10^{5}$	ND	ND
